# Machine learning for N-dimensional spatial reasoning tasks on the web

**DOI:** 10.3389/fbinf.2026.1694775

**Published:** 2026-03-16

**Authors:** Blake Moody, JieHyun Kim, Sanghyuk Kim, Daniel Haehn

**Affiliations:** Department of Computer Science, University of Massachusetts Boston, Boston, MA, United States

**Keywords:** artificial intelligence, computer vision, edge computing, genetic algorithm, machine learning, spatial reasoning, tracking

## Abstract

Spatial reasoning is essential for solving complex tasks in dynamic and high-dimensional environments. However, current training models for spatial tasks are computationally demanding and heavily reliant on human input. To address this gap, we present Snake-ML, a web-based simulation tool and proof-of-concept framework designed to demonstrate client-side training of spatial reasoning tasks. Snake-ML serves as an efficient and intuitive test bed for developing spatial navigation strategies in browser-based environments. We chose the snake game as our test bed because it is well suited for demonstrating spatial reasoning in low-dimensional visual spaces while remaining relevant to higher-dimensional tasks, compared to alternative methods. Through quantitative analysis, on the edge alone, Snake-ML achieves a 4.58× speedup in model inference. Additionally, we developed a direct TensorFlow.js GPU pipeline that achieves up to a 32× speedup in training time without any CPU/GPU synchronization. This pipeline has the potential to improve many edge-based AI visualization projects. Snake-ML shows potential for adaptability to complex spatial tasks, such as autonomous systems, robotics, and AI-driven environments. Our code and web-based simulation tool are publicly available.

## 1 Introduction

Spatial reasoning is a quality of intelligent beings that describes their ability to understand and navigate their environment by modeling relationships between objects and locations. This skill is crucial for interpreting proximity, orientation, direction, and movement in space. It plays a vital role in fields such as robotics, autonomous vehicles, virtual environments, and embodied artificial intelligence, where agents operate in dynamic, high-dimensional spaces. However, existing models require substantial computational resources, rely heavily on centralized infrastructures, and often struggle to generalize across dimensions. Recent studies on spatial cognition (Schaeffer et al., 2023) and high-dimensional signal processing (Kolouri et al., 2017) emphasize the need for frameworks that can efficiently encode and navigate high-dimensional spaces while maintaining learning stability. Our work is inspired by advancements in self-supervised spatial learning, such as the generation of grid-like representations without supervision (Schaeffer et al., 2023), and by generative techniques such as GANzzle++ (Talon et al., 2025), which solve spatial puzzles using latent assignments. Spatial data environments such as Eco-ISEA3H (Mechenich and Žliobaitė, 2023) demonstrate the value of structured, high-dimensional inputs for learning models. Meanwhile, work on optimal mass transport (Kolouri et al., 2017) and reinforcement learning for sensor positioning (Wang et al., 2019) underscores the importance of structured decision strategies across dimensions. Simulation-driven learning (Meng et al., 2025), geospatial AI for spatial modeling (Döllner, 2020), and robust high-dimensional modeling (Manigrasso, 2024) further confirm the value of combining vision, geometry, and learning^1^, ^2^.

**FIGURE 1:**
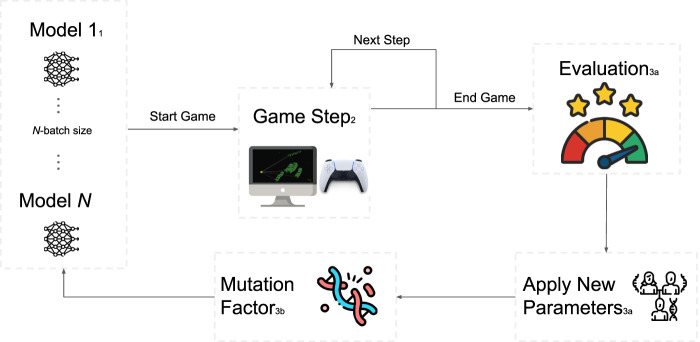
Real-time training loop in Snake-ML, implemented using the genetic learning algorithm (GA). 1) First, the population of snakes is initialized with random weights. 2) The games are then run in parallel and do not conclude until all of the games are over. 3a) The fitness scores of each snake candidate are evaluated, and the weights of the best snakes are passed onto the next-generation. 3b) A mutation factor is applied to encourage behavior exploration. 4) The process is repeated.

In response, we introduce Snake-ML, a web-based tool for training spatial agents in n -dimensional environments, as shown in Figure 1. Snake-ML generalizes the classic snake game to n dimensions, granting agents more degrees of freedom than in the original game, where the snake can only move forward, left, or right on a 2D game board. Modeled after this arcade classic, Snake-ML transforms gameplay into a spatial reasoning challenge where agents must navigate, avoid obstacles, and optimize scores in increasingly complex spaces. Our model utilizes advanced computer vision algorithms to avoid death and achieve the highest score. Our goal is to provide a clean, visual environment where learning progress can be tracked and interpreted, allowing both researchers and learners to understand spatial behavior in action. Snake-ML’s WebGL visualization, paired with client-side machine learning, allows for the study of how models explore optimal strategies for high-dimensional spatial tasks. In contrast to puzzle tasks such as GANzzle++ (Talon et al., 2025), the development of game strategies in Snake-ML is easier for human observers to interpret. A better understanding of model behavior can facilitate the design of more robust training algorithms that improve learning outcomes. Our system leverages unsupervised learning, inspired by recent advances in grid cell representation (Schaeffer et al., 2023), and uses vision-based control and collision detection strategies supported by geospatial and latent learning research (Döllner, 2020; Talon et al., 2025).

Snake-ML’s 3D visualization runs entirely in the browser via WebGL (Khronos Group, 2011) and ECMAScript (Ecma International, 1997), enabling scalable, privacy-preserving training directly on edge devices. Unlike most simulation-based spatial training frameworks that depend on cloud backends or proprietary infrastructure, Snake-ML runs in any modern browser with minimal setup, offering platform independence and low cost. Inspired by human-in-the-loop paradigms (Holzinger, 2016), its web-native design also supports intuitive experimentation and visualization for non-expert users.

## 2 Framework design

### 2.1 Implementation

#### 2.1.1 Genetic learning

Snake-ML uses a genetic learning algorithm to train the snakes. Genetic algorithms were introduced in the late 1980s. Genetic learning algorithms are suitable for edge training because they do not require a computationally expensive backward pass \citep{alam2020geneticalgorithmreviewsimplementations}. Backpropagation can be computationally expensive when the parameter space for optimization is high-dimensional (Kumari et al., 2024). This performance gain is critical because training on the edge has been shown, in some cases, to hinder hardware performance (Khouas et al., 2024, Pg.4). Genetic learning mirrors the animal kingdom, in which we select only the best-performing, fittest candidates to repopulate subsequent generations of training. In the case of multidimensional snakes, the selected candidates are the snakes.

**FIGURE 2:**
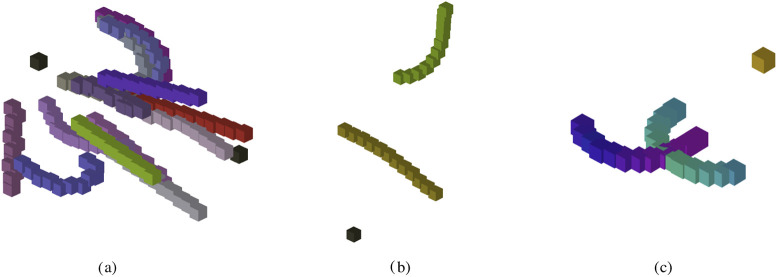
3D WebGL visualization. This figure showcases three currently available training visualizations. **(a)** Color-coded by individual snake candidate in the training episode. **(b)** The snakes are displayed as a heatmap, where green corresponds to high fitness reward at san instant in time and yellow represents low reward. **(c)** In higher spatial dimensions, snake’s position along its w-axis is represented in color space; w -positions of the snake’s body segments are mapped to its mesh’s hue.

Figure 2a assigns a unique RGB value to each candidate during a training episode. This helps distinguish better-performing candidates when they overlap with others. In the center, Figure 2b shows the current reward fitness at a specific instant in time. A green hue indicates greater reward for a snake candidate, and yellow indicates less or no reward. On the right, Figure 2c represents a training episode with four or more spatial dimensions. Hue is determined with a periodic function, with respect to the snake’s w -position $\left(hue=\frac{sin\left(w\right)+1}{2}\right)$ . Although two snakes appear to be overlapping, their differing hue values indicate that they are not.

#### 2.1.2 Model architecture

The snake model consists of two trainable feedforward layers with a Tanh activation function and a bias (see Table 1). The shape of each layer is dependent on the two hyperparameters: the number of snakes $B\in Z^{+}$ such that B≥1 and the number of spatial dimensions $N\in Z^{+}$ such that N≥2 .

**TABLE 1:** Model architecture summary. The batch size B and the number of dimensions N are variable during training and inference. FC refers to a fully connected layer.

Layer	Input shape	Output shape	Activation	Bias
FC	(B, 3N − 1)	(B, 24)	Tanh	Yes
FC	(B, 24)	(B, N − 1)	Tanh	Yes
Number of parameters			=48B(2N−1)	

**FIGURE 3:**
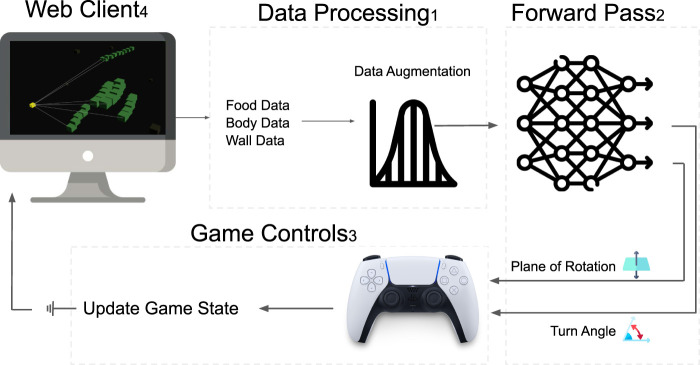
Behavioral loop in Snake-ML. 1) The snake model observes its environment via its food, body, and wall data and augments it after a change in the environment is made (refer to 2.1.3). 2) Data are passed into the network and output a turning angle and rotation plane. 3) With these control data, we apply an n-dimensional rotation matrix to the snake’s direction vector and apply the direction vector to its position. 4) The game state is rendered to the client. 5) The process is repeated.

We start the training by spawning a population of snakes in the game world, and they play the game independently until they fail. During each frame of the game, the snake collects input data about its position relative to the targeted food (Figure 3 left), the game boundary (Figure 3 middle), and segments of its tail (Figure 3 right). We feed the data into the network, which produces a control vector (i.e., boosted final score). We discuss the control vector in detail in Section 2.1.4.

#### 2.1.3 Data augmentation

We provide each snake with the position of its target food, the half-length of the bound box, and its position and direction. Since the relative Euclidean distance of the snake to the environment (food and bounds) is invariant to rotation and translation transformations (special Euclidean group (SE(N)) (Cederberg, 2010), we can augment the features of the snake’s environment; therefore, changes in distances become more predictable (or more formally, more equivariant) by providing the network with functional distance calculations related to the walls, food, and body (Bhardwaj et al., 2023; Finzi et al., 2020).

First, we augment the food position data. To make the food position invariant to the snake’s global rotation and translation, we can decompose the food’s displacement relative to the snake into a collection of signed angles along different planes of rotation. These planes of rotation are constructed with the basis vectors of the snake’s rotation matrix. Let ${\vec{r}}_{x}$ be the food’s position in global coordinate space. Let R be the snake’s rotation matrix, which stores information about its orientation in global coordinate space.

We calculate the displacement vector and project it to the snake’s local coordinate space, as presented in Equation 1:

$$\vec{v}=R{\left({\vec{r}}_{x}-{\vec{r}}_{p}\right)}^{T}.$$ (1)

We chose to measure the signed angle between v and u , where u is the first basis vector of our snake’s rotation matrix. This is also referred to as the “forward vector” of our snake because it is the direction our snake moves at each time step (Equation 2):

$$\vec{\theta }=atan2\left(u,v\right).$$ (2)

Second, we need to express the distance of the snake to the bounding box relative to its orientation. We achieve this by computing a ray that intersects with a plane containing a surface of the bounding box. The value of t represents the length of the ray from the snake’s position $r_{s}$ to its point of intersection with the plane. The ray has the same direction as the snake’s forward direction vector u . The equation for the ray x(t) is presented in Equation 3:

$$x\left(t\right)=r_{s}+tu.$$ (3)

The scalar value t is an orientation- and translation-invariant game state of our snake and is included in the input vector to our model. We derive Equation 3 for the bounding-box proximity along axis j . Let $n_{j}$ be a plane’s normal vector that lies on an axis of the global coordinate space. Let $r_{s}$ be the snake’s position in the global coordinate space (Equations 4, 5):

$$t_{j}=\frac{\text{sign}\left(u_{j}\right)L-r_{s,j}}{u_{j}},\;\text{for }\;u_{j}\neq 0,$$ (4)

$$\Delta t=\left[\begin{array}{c} t_{0}\\ \vdots \\ t_{n} \end{array}\right].$$ (5)

Using the same principle demonstrated for food position augmentation, we take the snake’s body-part positions in global coordinates, denoted by the matrix H . Then, we must compute a displacement for each body segment relative to the snake’s position. In addition, we project the displacement matrix to the local coordinate space $H^{\prime }$ (Equation 6):

$$H^{\prime }=R{\left(H-r_{p}\right)}^{T}.$$ (6)

Unlike the food, for which we want the snake to zero in on the food’s location, we want the snake to learn to avoid its body parts. Because of this, we avoid computing signed angles and instead compute local displacement vectors for each body segment of the snake. Our final displacement vector Δh represents the minimum component of all body segments along a particular axis in the snake’s local coordinate space. We do not concern ourselves with how close a snake is to each body segment. Instead, we focus on its closest body segment as it requires the fewest movement steps to result in a collision (Equation 7):

$$\Delta h_{\text{raw}}=\underset{\text{col}}{\min}\left(H^{\prime }\right).$$ (7)

Additionally, since distance in space has no bounds, we normalize using an L2 norm. We also avoid using an activation function on these model inputs (Equation 8):

$$\Delta h=\frac{\Delta h_{\text{raw}}}{\|\Delta h_{\text{raw}}{\|}_{2}}.$$ (8)

Among the three data augmentations shown above, the model’s input state is a concatenation of target angles $\vec{\theta }$ , boundary distances Δt , and body segment proximity Δh

#### 2.1.4 N-dimensional movement

To turn a snake in n-dimensions, we utilize the Aguilera and Pérez-Aguila algorithm for n-dimensional rotation (Aguilera and Pérez-Aguila, 2004). We compose an n-dimensional rotation by applying a series of rotations along different planes and then use the resultant rotation matrix to our snake’s current direction vector. Each value of the model’s output (control vector) corresponds to an angle θ on a plane of rotation for the snake. We do not want the snake to “barrel-roll” to avoid moves with no change in direction, so we exclude any planes whose normal vector parallels our snake’s direction vector. Furthermore, we want to preserve the handedness of our snake’s frame of reference after successive rotations, so we store not only the forward direction vector but also all basis vectors orthogonal to ${\vec{v}}_{0}$ . We apply the rotation to all stored basis vectors. We add the new, rotated direction vector to the snake’s current position vector to update the snake’s position. After the direction vector and all orthogonal basis vectors are rotated, we repeat the update process with the new position, direction, and basis vectors until the training conditions are met (all snakes die, maximum fitness is reached, etc.).

Our model understands its environment through its frame of reference. Therefore, its output features correspond to its frame of reference. We generate a rotation matrix to apply to the snake’s direction vector using a method for n-dimensional rotation. The snake records its position within the game space and its direction vector. The snake makes moves by turning its direction vector by a certain amount [theta] concerning a two-dimensional cross-section (two-plane) of the game space (Aguilera and Pérez-Aguila, 2004). For the snake game, the subsection is limited to the current direction vector of the snake $v_{f}$ and some arbitrary vector $v_{x}$ . This arbitrary vector $v_{x}$ and angle θ are determined by the control vector. In each game frame, we add the snake’s direction vector to its current position to obtain its next position. We continue this until all snakes either die or reach their maximum score. This process continues until all snakes either fail or achieve a predefined maximum score.

#### 2.1.5 Survival of the fittest

In each game frame, each snake has a choice rating from −1 to 1; the cumulative scores of snakes per game provide the best method for selecting the fittest snakes. Let $p_{i}$ be the current position of the snake, $p_{i-1}$ be the previous position of the snake, and $p_{f}$ be the position of the current target food. We measure the incremental fitness of a snake at timestep i by quantifying how close it has come to its goal of eating the food (Equation 9):

$$score_{\mathrm{current}}=\|{\vec{p}}_{\mathrm{prev}}-{\vec{p}}_{\mathrm{food}}\|-\|{\vec{p}}_{\mathrm{current}}-{\vec{p}}_{\mathrm{food}}\|.$$ (9)

Once all the snake games have ended, we evaluate the snake candidates by selecting pairings of parents for each current snake from a probability distribution based on the snakes’ relative fitness scores. In a crossover process, we derive a new model for each subsequent snake by randomly selecting individual values from either parent. After that, we mutate the newly created snake model by randomly adjusting different parameters. The probability of a parameter being mutated is called the mutation rate. The maximum magnitude that a parameter can be mutated is called the mutation factor.

## 3 Training on the edge

To enable edge model training, snake training batches were executed in parallel to minimize performance loss.

This project uses a JavaScript library called TensorFlow.js, which is a part of the popular ML Python library TensorFlow (Smilkov et al., 2019). The library uses WebGL and other widely adopted web frameworks, such as WebAssembly (Haas et al., 2017) and WebGPU (W3C GPU for the Web Working Group, 2021), to perform GPU operations on the client. WebGL texture data back the tensors in TensorFlow.js. This suggests that any modern computer, even without a dedicated GPU (although not recommended for performance reasons), can run WebGL and, by extension, TensorFlow.js. TensorFlow.js enabled us to compute entire training populations of snakes in batches, frame by frame, allowing viewers to visualize the model’s learning progression in real time with low latency.

In the case of rendering training simulations for all the snakes in real time, web workers can help prevent screen rendering from bottlenecking the training, or *vice versa* (Hickson, 2009). The main thread renders the game states using the popular WebGL framework Three.js (Cabello, 2010). Then, on a background thread, the trainer runs in a loop until the main thread requests that the worker stop the trainer. Although this prevents the client from freezing when running large models, specific performance issues, such as lower frame rates due to GPU-to-CPU synchronization, are addressed in subsequent sections.

## 4 Training on the cloud

For a valid performance comparison, we use a previous iteration of Snake-ML as the baseline performance metric for our edge framework. The older framework runs with the server training the models and clients and then connects to the server to receive a stream of model inference data, which is used to display the snake game for the viewer. The framework runs using PyTorch, a machine learning library that the OpenAI Gym library also utilizes. Similar to our edge framework, the cloud framework trains models using GA. However, it does not augment the environment data; instead, it passes the raw food, body, and wall data directly to the feedforward layers. The edge and cloud snake models share identical architectures, comprising two feedforward layers, Tanh activation functions, and a bias term.

## 5 Evaluation

To compare performance, we measured the time for a single update game simulation on both the edge and cloud frameworks. For benchmarking, we used an Asus Zephyrus G15 with an AMD Ryzen 9 5900HS (8-core, 3.3 GHz) CPU, 16 GB of DDR4 memory, and an NVIDIA RTX 3070 (8 GB GDDR6). Four hundred samples were collected across 20 different batch sizes, with 20 trials per batch size.

### 5.1 Performance

As shown in Table 2, the difference in the average update times between the edge and cloud frameworks increases as the batch size increases. Comparing both cases, we found that the edge framework achieves, on average, a 4.03× speedup over the cloud framework and up to a 4.58× overall speedup, as shown in Table 2. For the sake of this comparison, we executed the cloud framework on the same device as the client that renders the training, assuming negligible network latency. However, in practical applications, network latency is expected to be a more significant factor in cloud computing than edge computing (Lin et al., 2020).

**TABLE 2:** Performance improvement using CPU/GPU edge computing. Client-side training and inference improve the performance compared to cloud computing.

No. of snakes	Training time cloud (ms)	Training time edge (ms)	vs. Cloud speedup
300	430	107	4.02×
400	575	137	4.19×
500	722	169	4.28×
600	871	203	4.29×
700	1,016	244	4.17×
800	1,159	278	4.17×
900	1,300	318	4.09×
1,000	1,447	335	4.32×
1,100	1,600	362	4.42×
1,200	1,737	402	4.32×
1,300	1,878	425	4.42×
1,400	2,025	474	4.27×
1,500	2,174	497	4.37×
1,600	2,336	510	4.58×
1,700	2,461	538	4.58×
1,800	2,606	583	4.47×
1,900	2,766	617	4.48×
Average			4.03x
Max			4.58x

## 6 Unified WebGL context

One major limitation in rendering performance is how WebGL handles separate contexts. Although Three.js and TensorFlow.js utilize WebGL, they operate in individual contexts, indicating that their textures are stored in different locations on the GPU. As a result, transferring data between the training and rendering processes requires GPU-to-CPU-to-GPU communication, which causes overhead when rendering many snakes. This cost can be noticeable when the number of snakes to be rendered to the screen is large.

**FIGURE 4:**
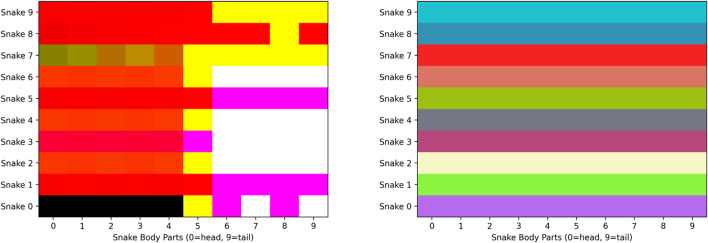
Game data loaded as textures in the WebGL shader program. TensorFlow.js tensors are represented by RGBA values on a WebGL texture. Above are the loaded textures for the snake body part positions (left) and colors (right). Each texture in the example encodes the positions of nine snakes, each with a maximum length of 10. Each row of each texture contains the snake body parts of an individual snake. The head of the snake is encoded as an RGBA pixel (4 × 32-bit floating point values), and subsequent parts of the snake are positioned and colored from left to right on the position and color textures, respectively. Each snake has uniformly colored body segments, and therefore, the colors on the color texture do not change along the x-axis.

We have developed a unified WebGL context (UWC). This GPU pipeline enables Snake-ML to initialize the visualization canvas and the TensorFlow.js GPU backend with a unified shared WebGL context. Once we achieve this, when performing inference with TensorFlow.js, we extract the WebGL texture backing the tensor and assign it to a new uniform variable. We then load that texture as a uniform in a WebGL shader program, and now, we can update the positions of snakes using the vertex shader and colors of snakes using the fragment shader (see Figure 4).

After running the same performance evaluation on Snake ML using the UWC, it achieves up to a 7.13× speedup compared to the standalone edge build and up to a 31.97× speedup compared to the original cloud build, as shown in Table 3. Although Three.js does not currently support loading external textures, we aim to develop a standalone Three.js library to set up a similar UWC using TensorFlow.js in the future. Given the broad adoption of Three.js and TensorFlow.js, a tool like this could have a considerable impact on the development community.

**TABLE 3:** Performance improvement using GPU-only edge computing**.** With a unified WebGL context (UWC) that keeps data in GPU memory, we achieve marked performance speedups compared to CPU synchronization and cloud training once more than 300 snakes are initialized.

No. of snakes	Training time cloud (ms)	Training time UWC (ms)	Cloud speedup
300	430	75	5.72×
400	575	79	7.28×
500	722	81	8.93×
600	871	78	11.16×
700	1,016	79	12.78×
800	1,159	76	15.26×
900	1,300	77	16.93×
1,000	1,447	83	17.53×
1,100	1,600	86	18.64×
1,200	1,737	83	20.98×
1,300	1,878	87	21.51×
1,400	2,025	89	22.74×
1,500	2,174	86	25.18×
1,600	2,336	93	25.16×
1,700	2,461	93	26.57×
1,800	2,606	103	25.40×
1,900	2,766	87	31.97×
Average			15.97x
Max			31.97x

## 7 Limitations

Although Snake-ML has demonstrated noteworthy improvements in AI training visualization, further research is needed on the model’s learning performance during edge training. However, genetic algorithms and other gradient-less optimization techniques have historically resulted in lower computational overhead. However, in theory, Snake-ML’s data augmentation strategies can be differentiated, and therefore, the benefits of backpropagation when implementing this data augmentation would be a relevant path for further research.

## 8 Conclusion

This article introduces a web-based framework for training and simulating models to play the snake game in n-dimensions. To demonstrate real-time policy optimization, we implement a genetic learning algorithm that trains the model progressively, over time, in test batches. Additionally, we perform heavy data augmentation as a pre-processing step before feeding the augmented data into our equivariant neural network. This approach results in a reduced overall neural network parameter size and notable hardware performance gains. In evaluating the hardware performance of the edge framework, we achieved an average 4.03× decrease in training latency compared to our previous cloud framework for training snake agents. By using pure WebGL for rendering, we also developed a unified WebGL context build of Snake-ML that avoids GPU-to-CPU synchronization between TensorFlow.js and our canvas. This was a noticeable improvement, resulting in a 31.97× speedup compared to the cloud build. Although edge training remains an underexplored approach for model development, our results demonstrate that edge computing offers marked potential for the future of AI systems.

Snake-ML demonstrates that high-dimensional learning, visualization, and optimization pipelines can be deployed entirely on the client—without cloud infrastructure—while remaining interpretable and performant. The client-exclusive training pipeline reduces major hardware barriers for individuals seeking to experiment with model training and real-time model visualizations. WebGL is supported by virtually every modern browser and operating system. Therefore, tools such as Snake-ML can provide a low barrier to entry, especially for students and researchers in under-resourced environments, and enable rapid prototyping without DevOps overhead.

### 8.1 Future work

#### 8.1.1 Generalizing framework

OpenAI’s Gym served as a considerable inspiration for this study. Building on the methods developed here, we aim to generalize the framework to enable broader applications, allowing users to easily implement their own spatial-reasoning training algorithms. In future work, we aim to extend the framework to handle more complex environments, including static and dynamic obstacles and a greater variety of bounding box geometries. We can also apply many techniques developed to optimize spatial reasoning models at a smaller scale to broader fields, such as robotics and autonomous navigation.

#### 8.1.2 Localized peer-to-peer federated learning

Federated learning (FL) is the technique in which multiple models train independently of each other, and their knowledge is aggregated in a centralized location (Yurdem et al., 2024). Training on the edge offers considerable security advantages for end users; however, can we leverage the benefits of collective learning without compromising edge security? In the future, we aim to explore this topic by investigating Bluetooth and other peer-to-peer (P2P) methods of data transfer to share knowledge between edge models within close device proximity.

## Data Availability

Publicly available datasets were analyzed in this study. This data can be found here: https://github.com/blayyyyyk/snake-ml.

## Footnotes

1 Code: https://github.com/blayyyyyk/snake-ml

2 Web-based tool: https://snakeml.moody.mx/
